# Specific Mutations Reverse Regulatory Effects of Adenosine Phosphates and Increase Their Binding Stoichiometry in CBS Domain-Containing Pyrophosphatase

**DOI:** 10.3390/ijms25115768

**Published:** 2024-05-25

**Authors:** Viktor A. Anashkin, Elena A. Kirillova, Victor N. Orlov, Alexander A. Baykov

**Affiliations:** Belozersky Institute of Physico-Chemical Biology, Lomonosov Moscow State University, Moscow 119899, Russiaorlovvn@belozersky.msu.ru (V.N.O.)

**Keywords:** allosteric regulation, cooperativity, cystathionine β-synthase domain, diadenosine tetraphosphate, enzyme regulation, isothermal calorimetry

## Abstract

Regulatory cystathionine β-synthase (CBS) domains are widespread in proteins; however, difficulty in structure determination prevents a comprehensive understanding of the underlying regulation mechanism. Tetrameric microbial inorganic pyrophosphatase containing such domains (CBS-PPase) is allosterically inhibited by AMP and ADP and activated by ATP and cell alarmones diadenosine polyphosphates. Each CBS-PPase subunit contains a pair of CBS domains but binds cooperatively to only one molecule of the mono-adenosine derivatives. We used site-directed mutagenesis of *Desulfitobacterium hafniense* CBS-PPase to identify the key elements determining the direction of the effect (activation or inhibition) and the “half-of-the-sites” ligand binding stoichiometry. Seven amino acid residues were selected in the CBS1 domain, based on the available X-ray structure of the regulatory domains, and substituted by alanine and other residues. The interaction of 11 CBS-PPase variants with the regulating ligands was characterized by activity measurements and isothermal titration calorimetry. Lys100 replacement reversed the effect of ADP from inhibition to activation, whereas Lys95 and Gly118 replacements made ADP an activator at low concentrations but an inhibitor at high concentrations. Replacement of these residues for alanine increased the stoichiometry of mono-adenosine phosphate binding by twofold. These findings identified several key protein residues and suggested a “two non-interacting pairs of interacting regulatory sites” concept in CBS-PPase regulation.

## 1. Introduction

The activities of many important enzymes, membrane transporters, and other proteins in all kingdoms of life are allosterically controlled via regulatory ligand binding to cystathionine β-synthase (CBS) domains. For instance, the human genome encodes 75 such CBS proteins, some of which are associated with hereditary diseases [1,2]. Most commonly, the regulatory ligands are various adenosine or guanosine phosphates [3,4,5,6]. The CBS domains are generally found in pairs in the protein sequence and form tightly associated “Bateman modules” in the ternary structure. The Bateman modules of two subunits further form four-domain disk-like “CBS modules” in homo-oligomeric proteins. Interestingly, the same enzyme may be regulated via CBS domains in some species, but lack them in other species. Furthermore, CBS domains can be deleted without loss of activity [7]. These findings suggest the utility of CBS domains as movable regulatory blocks for constructing regulated proteins. Because of the difficulty in obtaining structural data for full-size CBS proteins, presumably associated with the unusual flexibility of CBS modules, only limited information on the structural mechanism of CBS domain-mediated regulation is available.

**Figure 1:**
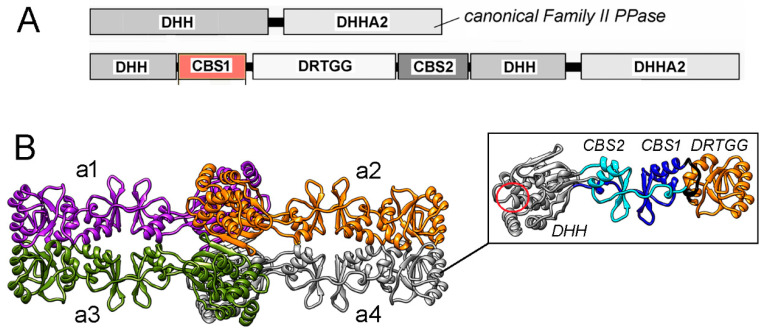
(**A**) Domain composition of canonical PPase and CBS-PPase of Family II. (**B**) 3D structure of tetrameric *dh*PPase without the DHHA2 domain suggested by low-resolution cryo-EM and molecular modeling [12]. The four subunits, a1–a4, are shown in different colors. The side panel shows separate subunit a4 with differently colored and labeled domains. The region of the active site retained in the truncated subunit is indicated by a red circle and is the location where the missing DHHA2 domain is attached. Panel B was reproduced from Zamakhov et al. [12] under the Creative Commons CC BY license.

Prokaryotic CBS domain-containing pyrophosphatase (CBS-PPase; EC 3.6.1.1), a typical CBS protein, belongs to Family II PPases, better known as a nonregulated, CBS domain-lacking form [8]. The latter is a dimer of identical subunits, each formed by DHH and DHHA2 domains, with an active site in between. CBS-PPase contains an ~250-residue regulatory insert of two CBS and one DRTGG domain in the catalytic DHH domain (Figure 1A). AMP and ADP inhibit CBS-PPase, whereas ATP, Ap_4_A, and other linear diadenosine polyphosphates (Ap_n_A, with *n* > 4) activate it by binding to the CBS domains [6,9,10]. The amino acid sequences of CBS domains reveal significant similarities between CBS-PPases and other CBS domain-containing proteins (CBS proteins), despite a considerable degree of variability. The DRTGG domain is absent in other CBS proteins and some CBS-PPases and hardly plays a significant role in their regulation.

Unlike the canonical Family II PPases, CBS-PPases form tetramers [11], which are stabilized by separate crosswise interactions of the catalytic and regulatory parts [12] (Figure 1B). The structure of tetrameric CBS-PPase from *Desulfitobacterium hafniense* (*dh*PPase) without the DHHA2 domain was obtained at 16 Å resolution by single-particle electron microscopy [12], whereas the structures of the dimeric regulatory part of homologous *Clostridium perfringens* CBS-PPase (*cp*PPase) in complexes with AMP (inhibitor) and Ap_4_A (activator) were determined at 2.3 Å by X-ray crystallography [13]. Although each CBS domain can bind a mono-adenosine phosphate and there are two CBS domains per subunit, the structures indicated the binding stoichiometry of one molecule of AMP per subunit or one molecule of Ap_4_A per dimer [10,13]. The latter ligand bridges two subunits by placing its two adenine moieties into the same sites occupied by AMP in each neighboring subunit.

CBS-PPase provides a good model for studying CBS domain-mediated regulation because it is easily accessible and stable, is differentially regulated by adenosine phosphates, including cell alarmones diadenosine polyphosphates, and its activity can be conveniently and precisely measured. In recent site-directed mutagenesis studies of *dh*PPase, we identified Arg276 and Arg295 in the CBS2 and Asn312 and Arg334 in DHH domains as having crucial roles in kinetic cooperativity (active site interaction) [14,15]. Another important finding was that the replacement of Arg295 or Asn312 by alanine reversed the effect of ATP from activation to inhibition [15].

In this study, we continued this line of research by seeking other residues that control the direction of the ligand effect (activation or inhibition). Our analysis of the available structural data predicted that such residues may belong to the CBS1 domain. Another goal of our study was to identify the structural determinants of the “half-of-the-sites” ligand binding stoichiometry and increase it in CBS-PPase.

## 2. Results

### 2.1. Selection of Residues for Substitution

**Figure 2:**
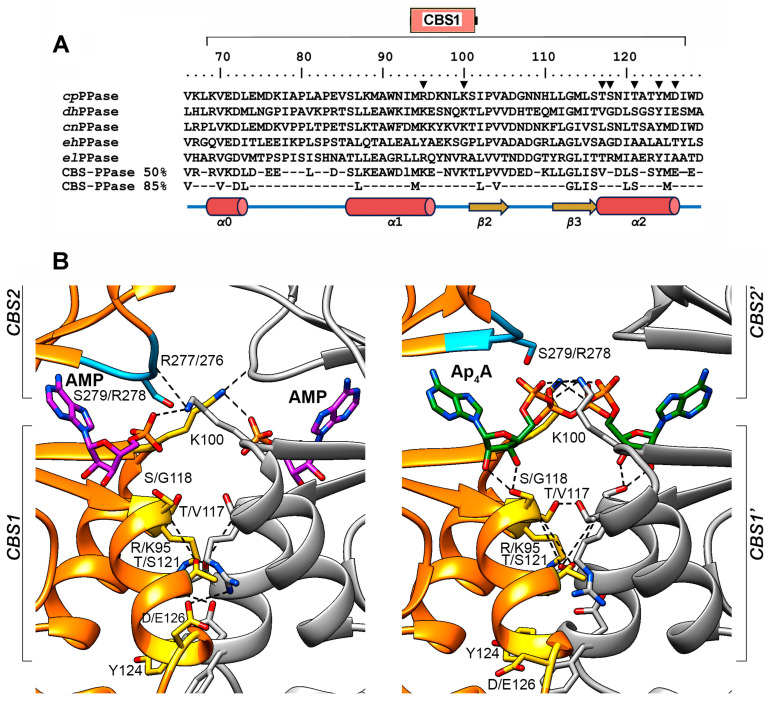
Selection of residues for substitution. (**A**) Partial sequence alignment of five CBS-PPases in the region of the CBS1 domain (residues numbers 69–127 indicated at *top*). The residue numbers shown are identical for *cp*PPase and *dh*PPase. The residues substituted in this study are marked with inverted triangles. The consensus sequences based on 50% and 85% identities in 457 CBS-PPases (KEGG GENES database of 28 March 2024) are shown below. *cp*PPase, *C. perfringens* CBS-PPase (KEGG ID: CPF_2312); *dh*PPase, *D. hafniense* CBS-PPase (Dhaf_1515); *cn*PPase, *Clostridium novyi* CBS-PPase (NT01CX_1928); *eh*PPase, *Ethanoligenens harbinense* CBS-PPase (Ethha_0050); *el*PPase, *Eggerthella lenta* CBS-PPase (Elen_0953). (**B**) AMP and Ap_4_A coordination in the regulatory S1 sites of *cp*PPase as determined by X-ray crystallography (PDB IDs 3L31 and 3L2B, respectively) [13]. The ribbon models of the two subunits are shown in different colors. The bound AMP and Ap_4_A molecules and the *cp*PPase amino acid residues corresponding to those substituted in *dh*PPase are shown as stick models; the *dh*PPase residues are indicated after the slash in the residue labels. RYRN loop is blue. Atoms forming an H-bond or ionic pair are connected by dashed lines.

CBS1 domains participate in three types of interactions: (a) with the CBS2 domain of the same subunit to form the Bateman module; (b) with the CBS1′ and CBS2′ domains of the neighboring subunit to form the four-domain CBS module; or (c) with the adenine, ribose, and phosphate groups of regulatory adenosine phosphates (Figure 2). Seven residues potentially important for these interactions were selected in the *dh*PPase CBS1 domain (Figure 2B), based on the structure of the regulatory part of homologous *cp*PPase [13] and the in-depth structural analysis of CBS proteins performed by Ereño-Orbea et al. [16]. Two of them (Lys100 and Tyr124) are identical in *dh*PPase and *cp*PPase, and the others are conservatively replaced in the pairs Lys/Arg95, Val/Thr117, Gly/Ser118, Ser/Thr121, and Glu/Asp126, where nonidentical residues found in the same position in *dh*PPase and *cp*PPase are separated by slashes. Six of these residues (except Gly/Ser118) form contacts with the neighboring subunit and six residues (except Lys100) belong to helical regions. Lys/Arg95 and Glu/Asp126′, belonging to different subunits, form two symmetrical ionic pairs at the subunit interface in the AMP complex. Lys100 and Ser121 form similar symmetrical intersubunit H-bonds with CBS2 Arg276′ and CBS1 Ser121′ residues, respectively. In addition, the Lys100 side chain neutralizes the charge of the α-phosphate group of the bound regulatory ligand. Val1/Thr117 and Tyr124 form a hydrophobic and stacking contact, respectively, with identical residues (Val/Thr117′ and Tyr124′) of the neighboring subunit. Residue 118 (Gly/Ser) is not involved in subunit contact but, like Val/Thr117, belongs to the ribose-phosphate binding motif G-h-h-S/T-x-x-D/N (residues 113–119; h is a hydrophobic residue, and x is any residue) [17]. This motif determines the permissible chemical structure of the substituent at the ribose O5′ atom of the regulating ligand [16].

All seven selected residues were replaced with alanine. In addition, Val117 was replaced by threonine, which is found in this position in >50% of CBS-PPases, including *cp*PPase (Figure 2A). Gly118 was additionally replaced by Ser, found in *cp*PPase (Figure 2A), and hydrophobic Ile and Met, found in S-adenosyl methionine-binding CBS domains [16]. All *dh*PPase variants were produced in a tag-less form in *Escherichia coli* and purified to apparent homogeneity using ion exchange and size-exclusion chromatography.

### 2.2. Effects of Substitutions on Tetramer Activity and Stability

**Figure 3:**
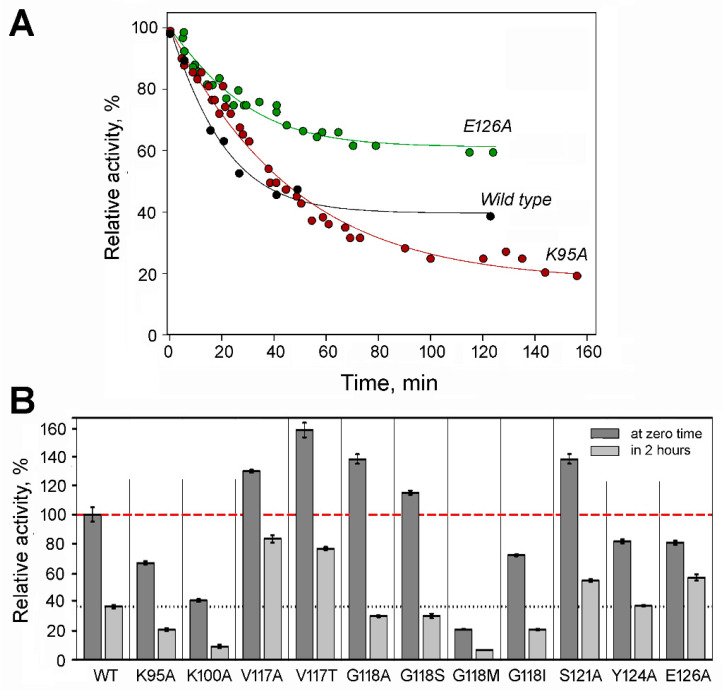
Specific activity and tetramer stability of *dh*PPase variants. (**A**) Representative time courses of activity for wild-type *dh*PPase and selected variants upon dilution at 25 °C. Stock enzyme solution containing 160–200 μM enzyme, 0.1 M Mops/KOH, pH 7.2, 2 mM MgCl_2_, and 0.1 mM CoCl_2_ was diluted into the same buffer to 0.2 μM enzyme. Aliquots were withdrawn in time, and activity was measured using the standard procedure. The lines were created using the best-fit parameter values found in Table 1. Activity extrapolated to zero time by the fitting procedure (*A*_T_) was taken as 100% for each curve (actual values are found in Table 1). (**B**) Specific activities before dilution and after equilibration in the diluted state. Wild-type *dh*PPase activity before dilution (590 ± 30 IU/mg) was considered 100% for all variants. The bars show standard deviations of the mean in three independent measurements. The red dashed and black dotted lines indicate wild-type *dh*PPase activity levels before and after dilution, respectively.

**Scheme 1:**
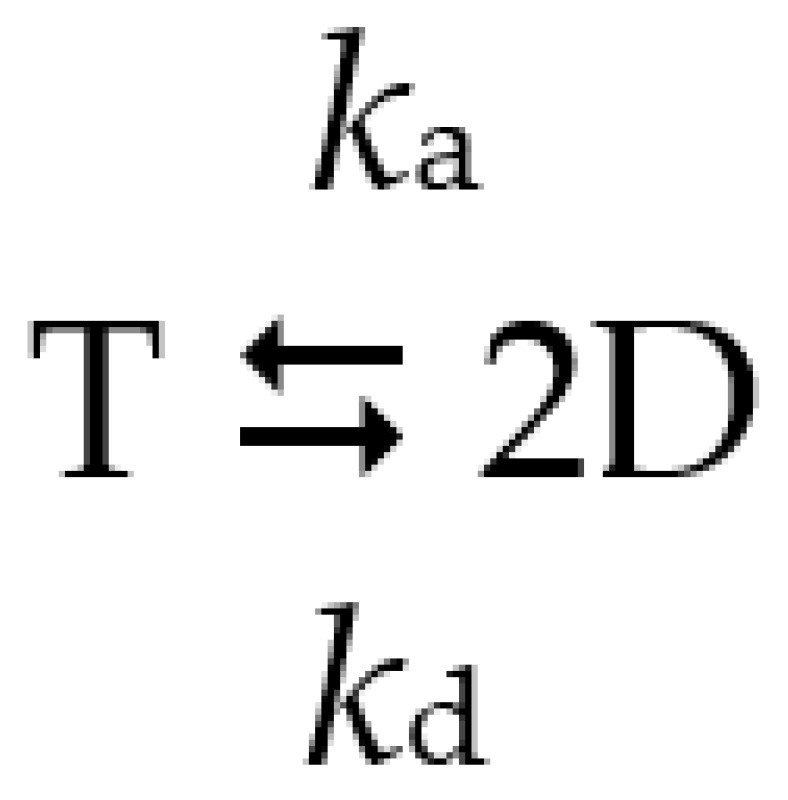
Reversible dissociation of the tetrameric enzyme into dimers. T is active tetramer, D is inactive dimer, *k*_a_ and *k*_d_ are the rate constants of association and dissociation.

The tendency to dissociate into inactive dimers in diluted solutions complicates the activity assay of *dh*PPase. Similar to the wild-type enzyme [11], dissociation was a relatively slow process and did not occur significantly during the time of the activity assay (2–3 min) but was evident in preincubated diluted stock solutions. Therefore, the time courses of enzyme activity upon dilution were measured to estimate tetramer stability and activity (Figure 3A). All variants were fairly active in PP_i_ hydrolysis and demonstrated a first-order transition to a new equilibrium with lower tetramer content and, consequently, lower activity (Figure 3B). The time courses were analyzed in terms of the equilibrium in Scheme 1, as described previously [11], to derive the values of *k*_a_, *k*_d_, and activities at zero and infinite times.

As Table 1 highlights, the substitutions generally had a moderate effect on tetramer activity. The effect varied from a 4.8-fold decrease for the G118M variant to a 1.4-fold increase for the V117T variant. The effects of the substitutions on the rate constants for the reversible dissociation were more pronounced and decreased both *k*_d_ and *k*_a_, except for the E126A variant, for which the *k*_a_ slightly increased. The effects on *k*_a_ generally prevailed, resulting in tetramer destabilization in terms of the equilibrium dissociation constant *K*_d_ = *k*_d_/*k*_a_, except for the V117T and E126A variants, which were more stable than the wild-type enzyme. Based on the *K*_d_ values obtained, the percentage of dissociated tetramer in the stock enzyme solutions before dilution in the experiments for Figure 3A and similar did not exceed 10% for the least stable variants (with Lys100 and Gly118 substituted).

### 2.3. Regulation of dhPPase Variants by Adenosine Phosphates

**Figure 4:**
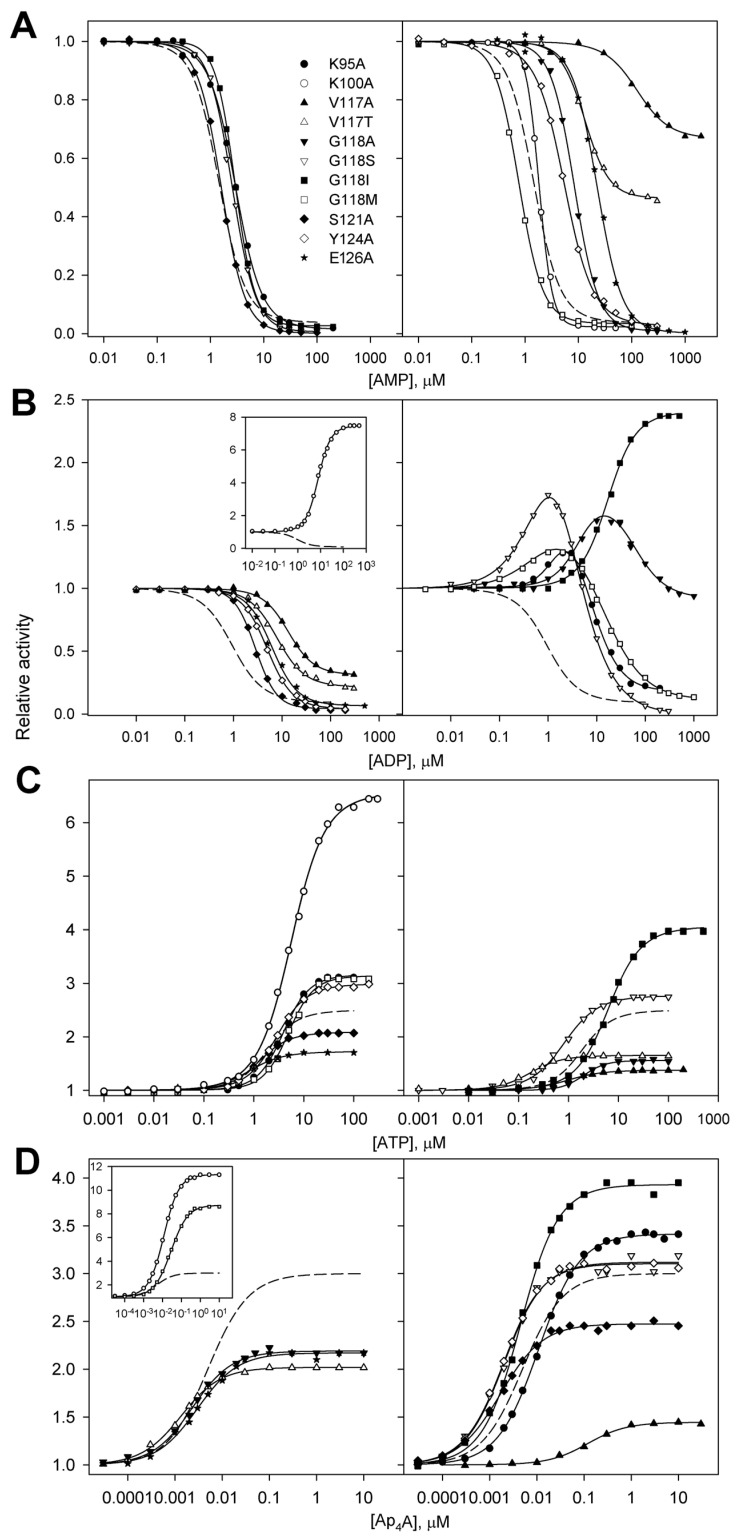
Effects of AMP (**A**), ADP (**B**), ATP (**C**), and Ap_4_A (**D**) on the activities of 11 *dh*PPase variants. Activity measured without adenosine phosphates (*A*_0_ in Equations (1) and (2)) was taken as unity for each variant. The symbols used are detailed in the top panel. The insets show the data for variants with the largest effects. The reaction of PP_i_ hydrolysis was initiated by adding enzyme pre-incubated in stock 0.8–3.2 µM (0.05–0.2 mg/mL) solution to the assay mixture containing 0.1 M Mops/KOH buffer, pH 7.2, 5 mM MgCl_2_, and 140 µM PP_i_ (yielding 50 µM MgPP_i_ complex). The solid lines show the best fits of Equations (1) or (2). The wild-type data (dashed lines) are from previous publications [9,10].

The effects of four physiological modulators, including AMP, ADP, ATP, and Ap_4_A, were tested with the *dh*PPase variants (Figure 4A–D). The first two inhibit the wild-type enzyme, whereas the last two activate it [9,10]. Furthermore, other linear diadenosine polyphosphates, Ap_n_A, with n = 3, 5, and 6 also activate *dh*PPase [10]. Thus, there is a correlation between the phosphate chain length in the CBS-PPase regulator and the direction of the effect—inhibition or activation. As the border lies between two and three phosphate groups, changes in the residues that sense polyphosphate length might shift this border and confer activation by ADP or inhibition by ATP to CBS-PPase.

In accordance with these expectations, only ADP inhibition was reversed in the six variants obtained by substituting three residues: Lys95, Lys100, and Gly118 (Figure 4B). The effects were, however, not uniform. While the activities of the K100A and G118I variants monotonically increased to a constant level up to 500 µM ADP concentration, four other variants demonstrated a bell-shaped dependence with activity finally dropping to ~1% (G118S), ~10% (G118M), 20% (K95A), or 90% (G118A) at the highest ADP concentrations.

In contrast, the effects of AMP, ATP, and Ap_4_A on the *dh*PPase variants were qualitatively similar to their effects on the wild-type enzyme—AMP inhibited the enzymes, whereas ATP and Ap_4_A activated them. However, there were important quantitative differences. Thus, the V117A and V117T variants demonstrated increased residual activity at saturating AMP levels (Figure 4A). Interestingly, the same effect was observed with these two variants in ADP inhibition (Figure 4B), and they additionally demonstrated decreased activation by ATP and Ap_4_A compared with the wild-type enzyme (Figure 4C,D). For the three variants (V117A, V117T, and E126A), AMP profiles were markedly shifted to higher AMP concentrations, indicating weaker ligand binding. For the K100A variant, the AMP profile was much steeper than those for all other enzyme forms, indicating increased binding cooperativity. The K100A variant surpassed all other enzyme forms in terms of the degree of activation by ATP, and the V117A variant demonstrated the highest sensitivity to low ATP concentrations. Two substitutions (K100A and G118M) increased activation by Ap_4_A to 9–11-fold, and one (V117A) markedly suppressed the activation by this ligand in terms of both binding affinity and the degree of activation.

For quantitative comparison, the monotonic dose dependences were analyzed using a rearranged Hill equation [18]:

(1) $$v=\frac{A_{1}+A_{0}{\left(\frac{K}{\left[N\right]}\right)}^{h}}{1+{\left(\frac{K}{\left[N\right]}\right)}^{h}},$$

where *A*_0_ and *A*_1_ are activities at zero and infinite ligand (N) concentrations, respectively, *K* is an apparent binding constant [*v* = (*A*_0_ + *A*_1_)/2 at [N] = *K*], and *h* is the Hill coefficient. This formal type of analysis was preferred to using various models of cooperativity [18] because it is simple, does not depend on structural assumptions, and nevertheless provides useful characteristics of binding cooperativity, the Hill coefficient. This parameter depends on the strength of the interaction between the binding sites and their number, and is unity for non-cooperative binding, less than unity for negative cooperativity, and greater than unity for positive cooperativity. In the latter case, the Hill coefficient may approach but never exceeds the number of interacting binding sites [18]. Although the Hill coefficient is related to free energy changes in successive binding events, such analysis requires additional information [19]. One should also keep in mind that *K*_N_ values are only approximate average estimates of true binding constants, which differ in different binding events because of the cooperativity involved in monoadenosine phosphate binding to CBS-PPase [9].

The parameter values obtained by fitting Equation (1) with the program Scientist (MicroMath) and listed in Table 2 support the conclusions made above based on the visual inspection of Figure 4. The K100A variant demonstrated an extraordinarily high Hill coefficient of 3.5 in AMP inhibition, indicating the presence of at least four strongly interacting binding sites for this ligand. In all other cases, the *h* value did not significantly exceed 2.0. The V117A substitution had the greatest effect on the binding affinity (decreased it) for all adenosine phosphates, except ATP.

To analyze the bell-shaped dependences describing the ADP effects on the four variants, an extended “Hill-type” equation was derived:

(2) $$v=\frac{A_{1}+A_{0}{\left(\frac{K_{1}}{\left[N\right]}\right)}^{h1}+{A_{2}(\frac{\left[N\right]}{K_{2}})}^{h2}}{1+{\left(\frac{K_{1}}{\left[N\right]}\right)}^{h1}+{\left(\frac{\left[N\right]}{K_{2}}\right)}^{h2}}$$

This equation contains two binding constants and two Hill coefficients, implying binding of ≥*h*_1_ activating and ≥*h*_2_ inhibiting ligand molecules; the activity *A*_2_ refers to the saturating concentration of the ligand; and *A*_1_ is the activity of the enzyme with only the activating ligand bound. The parameter values obtained using Equation (2) for the four variants are listed in Table 3. Interestingly, of the four Gly118 replacements, Gly/Ala had the largest effect on the binding affinity (*K*_1_ and *K*_2_), clearly indicating that the side chain size is not the key parameter for this residue position. The *h* value of 0.9–1.1 for the ascending and descending parts of the ADP profiles in the G118S and G118M variants suggests that they result from independent binding of two ADP molecules. In other words, these substitutions cancel ADP-binding cooperativity. In contrast, the cooperativity was retained in two other variants with bell-shaped profiles (Table 3), which is only possible if the number of interacting sites increases above two. Thus, either the network of interactions involves all four binding sites present in the wild-type enzyme, or new site(s) appear in the K95A and G118A variants.

S-Adenosyl methionine regulates cystathionine β-synthase by binding to its CBS domains [16], which contain Met in the position corresponding to *dh*PPase Gly118. However, this adenosine derivative had no effect on the activities of wild-type *dh*PPase and its G118M and G118I variants. Thus, it appears that these substitutions alone were not enough to confer the ability to accommodate S-adenosyl methionine to *dh*PPase CBS domains.

### 2.4. AMP/ADP and ADP/Ap_4_A Competition in Variants with Unusual ADP Effects

The AMP and, presumably, ADP binding stoichiometry is one molecule per Bateman module [10,13] and, hence, four molecules per tetramer, all of which appear to be inhibitory in the wild-type enzyme. Two replacements (K100A and G118I) apparently converted all ADP-binding sites into activating. In the four variants demonstrating bell-shaped ADP profiles (K95A, G118A, G118S, and G118M), only part of the binding sites became activating ones. Alternatively, the latter substitutions might result in the appearance of additional ADP-binding sites with an activating effect on the enzyme.

**Figure 5:**
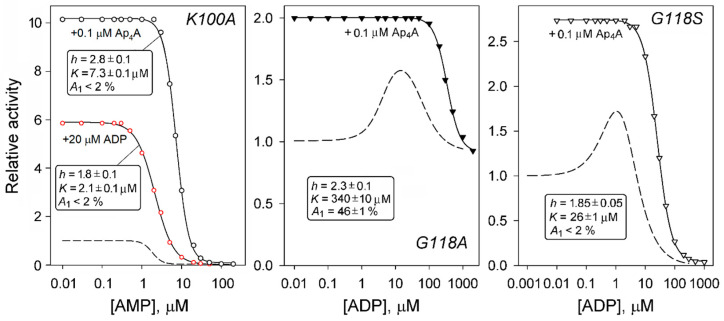
Competition between adenosine phosphates in selected *dh*PPase variants. The dashed lines show the activity dependence on the adenosine phosphate indicated on the abscissa and are taken from Figure 4. The solid lines with points show the same dependence measured in the presence of a fixed concentration of the adenosine phosphate indicated in the curve label. The parameter values estimated for the latter dependence using Equation (1) are shown in a box on each panel. The activities measured in the absence of any adenosine phosphate were taken as unity for all curves in each panel.

Two types of experiments were conducted to select between these alternatives. First, the binding stoichiometry was indirectly estimated by measuring the competition between AMP, ADP, and Ap_4_A in the activity assay (Figure 5). In these experiments, activity was measured at an increasing concentration of one ligand (AMP or ADP) and a fixed concentration of the other ligand (ADP or Ap_4_A). If the effects of the two ligands were independent of each other, the activity profiles would shift upward due to activation by ADP and Ap_4_A, without changing their shapes. This is clearly not the case. Moreover, when the dependences in Figure 4 for the “two-ligand” systems were analyzed with Equation (1), the Hill coefficient and the binding constants differed significantly from the values found in Table 2 and Table 3 for the corresponding “one-ligand” dependence. For the K100A variant, Ap_4_A and ADP decreased the Hill coefficient for AMP inhibition from 3.5 to 1.8 and 2.8, respectively. This is an expected behavior because the competing ligands (Ap_4_A and ADP) bind with no or lower cooperativity than AMP. This also explains the only moderate effects of ADP and Ap_4_A on the value of *K* for AMP (Figure 5 and Table 2).

In the G118A and G118S variants, Ap_4_A eliminated the activating effects of ADP and shifted the inhibition part of the bell-shaped profiles to larger ADP concentrations (Figure 5). These observations are consistent with Ap_4_A competition for both ADP binding sites responsible for the bell-shaped appearance of the profiles in its absence.

### 2.5. Measurements of Adenosine Phosphate Binding by Isothermal Titration Calorimetry (ITC)

**Figure 6:**
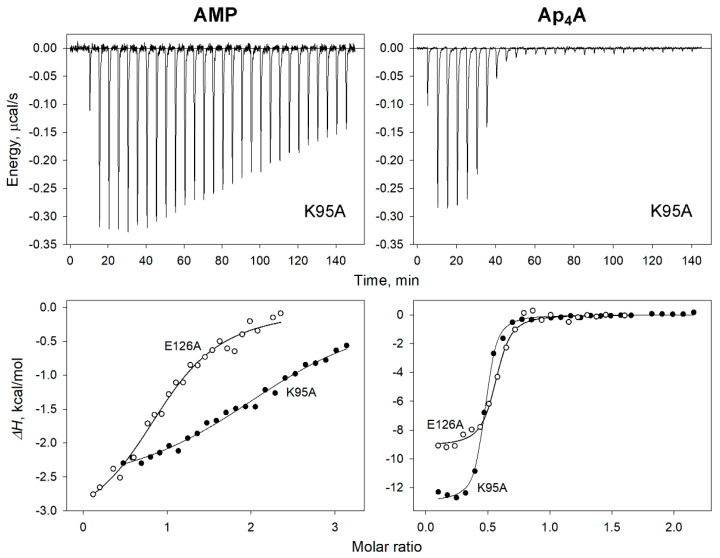
ITC measurements of adenosine phosphate binding to *dh*PPase variants. (**Top**) Typical raw data for successive injections of AMP or Ap_4_A into 8 μM solution of the K95A variant. (**Bottom**) Integrated heats for selected variant titrations by AMP (**left**) and Ap_4_A (**right**) after correction for dilution. The lines show the best fits of the single-binding-site model.

The binding stoichiometry of the adenosine phosphates was also estimated directly by isothermal titration calorimetry. In these experiments, enzyme solutions were titrated with increasing concentrations of the four ligands and enthalpy changes were recorded after each ligand addition (Figure 6, top panels). The bottom panels of Figure 6, show typical titration curves obtained for several *dh*PPase variants, and Table 4 summarizes all the information derived from the curves by fitting a simple binding equation with *n* binding sites per subunit. All the binding reactions studied were exothermic (Δ*H* < 0). The binding constant *K*_N_ was another variable parameter in these fittings; however, its estimated value should be considered as only a rough average estimate because monoadenosine phosphate binding to CBS-PPase involves positive cooperativity [9]. This also refers to the *T*Δ*S* values calculated from the *K* and Δ*H* values.

Most importantly, these data identified three variants with doubled binding stoichiometry *n* for all monoadenosine phosphates. Not surprisingly, these variants demonstrate unusual activity profiles in Figure 4. One of them (K100A) bound AMP with an unusually high positive cooperativity (Table 2), and two others (K95A and G118A) demonstrated bell-shaped profiles with ADP. Quite interestingly, two variants (G117S and G117M), which also showed bell-shaped profiles with ADP, nevertheless, demonstrated “normal” binding stoichiometry for all monoadenosine phosphates. In contrast, no significant changes were detected in the value of *n* for Ap_4_A binding to all variants (Table 4).

## 3. Discussion

CBS-PPase regulation by adenosine phosphates has six phenomenological characteristics: direction of the effect (activation or inhibition) and its size (regulation “intensity”), ligand binding stoichiometry, affinity, and cooperativity, and tetramer stability (its dissociation abolishes activity). In what follows, we consider the identities of the amino acid residues that control these characteristics (Table 5), speculate on the possible structural mechanisms of this control, and compare the obtained information with that reported for other enzymes regulated via CBS domains.

CBS-PPase is differentially regulated by adenosine phosphates—AMP and ADP inhibit it, whereas ATP, cAMP, and Ap_4_A activate it [9,10,20]. Apart from the polyphosphate chain length, three residues of the CBS1 domain appear to control the direction of the effect: Lys95, Lys100, and Gly118. Of these, Lys100 appears to be the most important, as its substitution by alanine completely reversed the ADP effect from inhibition to activation, whereas, in the K95A and G118A variants, ADP remained inhibitory at high concentrations (Figure 4). The remaining inhibition was, however, much less pronounced in the G118A variant than in the wild-type enzyme and simply counterbalanced the activation effect observed at low ADP concentrations (Table 2 and Table 3).

Similar effects have been previously observed with this and other CBS proteins. Thus, Arg/Ala and Asn/Ala replacements in the CBS2 domain of *dh*PPase converted ATP from an activator to an inhibitor [15]. Two substitutions in the CBS2 domain of *Moorella thermoacetica* CBS-PPase converted AMP from an inhibitor to an activator [21]. A similar modulation of the regulation of cystathionine β-synthase by S-adenosyl methionine [7] and AMP-dependent protein kinase by AMP and ATP has been reported [4,22]. Interestingly, the Arg residue of AMP-dependent muscle protein kinases, analogs of CBS-PPase Lys100, is the site of a natural pathogenic mutation [22,23,24]. In human cystathionine β-synthase, this position is also mutagenic but is occupied by Asp [25].

In the absence of the structure of the CBS-PPase complex with ADP, the structure of the regulatory part with bound AMP (Figure 2B) provides some clue to the reversal of the ADP effect in the K100A variant. In the wild-type enzyme, the pyrophosphate moiety of ADP likely also interacts with the Lys100 NH_3_^+^ group, and its removal should shift ADP upward to a nearby Ser279 hydroxyl group (Arg278 guanidino group in *dh*PPase). This shift is expected to convert the CBS2 domain into the “open” conformation found in the complex with the activator Ap_4_A, wherein such transition is mediated by a conservative RYSN /RYRN loop encompassing Ser279/Arg278 [13]. This transition and, hence, activation cannot occur with AMP because its short phosphate chain cannot reach Ser279/Arg278 but is permitted with ATP, which activates the CBS-PPase.

The dual activation/inhibition effect of ADP on the G118A/S/M variants needs a different explanation. Notably, there is a correlation between the ADP effect on activity and residue 118 sidechain size: both canonical sites are inhibitory if this residue is Gly, one site becomes activating in the Gly/Ala, Gly/Ser, and Gly/Met variants, and both sites are activating in the Gly/Ile variant. The presence of a residue with a side chain in this position may dictate the adenosine moiety to bind in the conformation adopted in the activated Ap_4_A complex in Figure 2B, allowing a similar partial “opening” of the CBS2 domain via the RYRN loop. However, because of electrostatic repulsion, the second ADP molecule cannot adopt the same conformation and triggers the conversion of both ADP molecules into the “AMP” conformation with concomitant inhibition. This conversion is apparently prevented by the large side chain in the G118I variant, making ADP activator at both sites.

Interestingly, the two Ala variants that demonstrated bell-shaped activation/inhibition profiles with ADP exhibited a twofold greater ADP-binding stoichiometry (Table 4), indicating the appearance of an additional binding site. This site accommodated all mono-adenosine phosphates and was likely the pseudosymmetry-related S2 site, “silent” in the wild-type enzyme. Notably, a binding stoichiometry greater than one per Bateman module has also been reported for four CBS2 domain variants of *M. thermoacetica* CBS-PPase [20] and authentic CBS domain-containing proteins [26,27,28,29].

The localization of the additional binding site was confirmed by AMP docking experiments with an AlphFold2-generated model of the regulatory part of the K100A variant of *dh*PPase (Figure S1). AMP docked to both the ‘canonical” S1 site occupied in Figure 2B and the “new” S2 site in this variant (Figure S1), with the values of the scoring function being 7.0 and 7.2 kcal/mol, respectively, i.e., very similar. Notably, the S2 site is functional in other CBS proteins. Thus, both sites are occupied by adenosine derivatives in the crystal structures of IMP dehydrogenase [3] and AMP-dependent protein kinase [29,30]. Moreover, the structures of cystathionine β-synthase [31] and chloride channel ClC-5 [32] contain the regulating adenosine derivative in only S2 site.

One could have speculated that ADP activates the variant *dh*PPase by binding to this “new” site and inhibits it by binding to the “canonical” site found in the wild-type enzyme (Figure 2B). This explanation was, however, ruled out by the bell-shaped dependence of activity on [ADP] for the G118S and G118M variants (Figure 4) that have no additional ADP-binding site (Table 4). In the latter variants, the observed activation and inhibition clearly result from ADP binding to the canonical sites—ADP activates the enzyme when bound to one site but inhibits when bound to both sites of the Bateman module, as discussed above. Consistent with this interpretation, the Hill coefficient is close to unity for both the activation and inhibition parts of the activity profiles for the G118S and G118M variants. In the presence of Ap_4_A, ADP only inhibits these variants because ADP displaces the Ap_4_A molecule (a better activator) occupying both canonical sites.

Because of the inherent cooperativity and the variable value of the Hill coefficient, the *K* value should be considered as only a rough estimate of the binding affinity in the case of mono-adenosine phosphates. In contrast, Ap_4_A binding is non-cooperative, and the determined *K* values report the binding affinities of the *dh*PPase variants for this ligand. The largest effects on Ap_4_A binding were observed upon Val117 substitution—increasing residue polarity (V to T substitution) stabilized the complex, whereas decreasing side chain volume (V to A substitution) destabilized the complex (Table 2). These findings emphasize the role of subunit interaction in CBS-PPase regulation. The fact that Val117 forms a hydrophobic interaction with the same residue of the partner subunit apparently doubled the effects of the Val117 substitutions. Val117 was also important for mono-adenosine phosphate binding, and the effects of its substitutions were opposite for complexes with AMP or ADP (destabilization) and ATP (stabilization).

The effects of E126A substitution were qualitatively similar. It should be noted that the *h* values were similar for the wild-type enzyme and the Val117 and E126A variants, justifying direct comparisons of the *K* values. Tyr124 also contacts its counterpart Tyr124′ of the neighboring subunit and apparently stabilized binding of only mono-adenosine phosphates in terms of *K*, the effect being larger for AMP and ADP (Table 2).

Wild-type *dh*PPase binds four molecules of the mono-adenosine phosphate ligands per tetramer in a positively cooperative manner, with a Hill coefficient between one and two (References [9,10] and Table 2). In the limiting case of positive binding cooperativity, the Hill coefficient could reach four, which is at least twice as high. A likely explanation of this difference is that the binding sites interact within the four-CBS-domain structure shown in Figure 2B, but not with the distantly located sites of two other Bateman modules (Figure 1B). The lack of cooperativity in the Ap_4_A binding by the wild-type and all *dh*PPase variants (Table 2) provides strong support for this idea. Each Ap_4_A molecule binds to two adjacent Bateman modules (Figure 2B) in the subunit pairs a1-a3 and a2-a4. In other words, the binding sites appear to be structurally and functionally organized in pairs and interact with each other only within these otherwise independent pairs.

The K100A variant demonstrated remarkably high cooperativity in AMP binding (*h* = 3.5). Consistent with the concept of “two non-interacting pairs of interacting regulatory sites”, this substitution unmasked an additional binding site in each subunit (Table 4), increasing to four of the total number of sites in two interacting Bateman modules. AMP binding to any of these sites markedly increases the binding affinity of the other sites; thus, at equilibrium, all Bateman modules are predominantly in a ligand-free form or in a complex with four AMP molecules, with a low content of intermediate forms.

Why did cooperativity not increase with ADP and ATP in the K100A variant and with any adenosine phosphate in the K95A and G118A variants (Table 2) despite the increased binding stoichiometry (Table 4)? Additional phosphate groups apparently constitute a steric constraint for ligand binding to the additional site, making it less favorable in terms of the free energy of the system. As a result, sequential binding of ligand molecules becomes less favorable and results in the accumulation of appreciable amounts of intermediate complexes and, hence, decreased cooperativity. The lack of a significant increase in the cooperativity upon the K95A and G118A substitutions indicates the importance of the ionic pair formed by Lys95 and the conformational freedom around Gly118 for the interaction between four binding sites in four adjacent CBS domains. In the G118S and G118M variants, cooperativity is completely lost because of the increased steric constraints imposed by bulky side chains, further signifying the importance of Gly118 for site interaction in the Bateman module pairs.

As six of the seven substituted residues are involved in subunit contacts, most substitutions decreased tetramer stability to approximately the same extent (*K*_d_ increased from 0.7 to 4.0–6.8 µM). Because partial dissociation of the tetramer can occur under physiological conditions and the dissociated form (presumably dimer) is inactive [11], this phenomenon may contribute to CBS-PPase regulation. In two variants (V117T and Y124A), the tetramer retained its stability, whereas V117A and E126A substitutions stabilized it (Table 1). The smaller size of the side chain in the substituting Ala and, hence, a closer subunit contact in the V117A variant apparently explains its stronger subunit interaction. The effect of the E126A substitution was unexpected because Glu126 and Glu126′ form intersubunit contacts with Lys95′ and Lys95, respectively, whose substitution by Ala destabilized the tetramer (Table 1). A similar asymmetry was noted above in the effects of Lys95 and Glu126 substitutions on the activity modulation by ADP. This asymmetrical behavior of the ion pair constituents may be rationalized in terms of the formation of a surrogate Asp130′—Lys95 ion pair in the E126A variant. This is, however, only a hypothesis and needs to be verified by structural studies.

The regulating adenosine phosphates can change the activity of the wild-type *dh*PPase from 3.7% (AMP bound) to 300% (Ap_4_A bound) (Table 2), i.e., 80-fold. All substitutions of most residues increased this dynamic range to 250–600, whereas the Val117 substitutions decreased it to 2.2–4.4. The same applies to the AMP/ATP pair because the activating effects of ATP and Ap_4_A are very similar and differ 2–3-fold with only K100A and G118M variants, decreasing the dynamic range by the same factor. Because PPase activity controls the concentration of pyrophosphate, the key regulator of biosynthesis [33], it would be interesting to determine in future studies the effects of the CBS-PPase mutation on the growth characteristics of their host bacterial species.

In summary, the results of this study define the role of the CBS1 domain in CBS-PPase regulation by adenosine derivatives. Our findings have demonstrated for the first time that by modifying specific amino acid residues in this domain, one can control the functionality of two binding sites for adenosine derivatives in the Bateman module, increase the size of their regulating effect, reverse it, and make it more sensitive to changes in regulating ligand concentration. This information adds to the notion that CBS domains are promising transmissible blocks for engineering proteins sensitive to the distribution of various adenosine phosphates, i.e., cell energy status. However, the implementation of this idea will require a broader understanding of the regulation mechanism associated with CBS domains. Most importantly, further exploration should be undertaken to investigate the way by which the regulating signal reaches the distantly located active site.

## 4. Materials and Methods

### 4.1. Materials

Wild-type *dh*PPase (UniProtKB: B8FP42) and its variants were produced in *E. coli* BL21 cells transformed with the pET-42b Novagen vector (Sigma-Aldrich Co, St. Lous, MO, USA) carrying the corresponding genes. All mutations in the CBS1 domain part of the *dh*PPase gene were performed using overlap extension PCR with Phusion DNA polymerase (Thermo Fisher Scientific Baltics UAB, Vilnius, Lithuania). The forward and reverse primers are listed in Table S1. The protein isolation procedure [11] included cell disruption by freezing/thawing, ion exchange chromatography on DEAE Toyopearl 650M (TOYO SODA MFG, Tokyo, Japan), and size exclusion chromatography on Superdex 200 (GE Healthcare Bio-Sciences AB, Uppsala, Sweden), with absorbance monitoring at 280 nm. The final protein preparations were stored frozen in the elution buffer used at the gel filtration step (0.1 M Mops/KOH, pH 7.2, 2 mM MgCl_2_, 0.1 mM CoCl_2_, and 150 mM KCl). The purity of the isolated proteins, as estimated by SDS–PAGE [34] with Coomassie staining, was >90%. Protein concentrations in milligrams per milliliter were determined spectrophotometrically using the extinction coefficient *A*_280_^0.1%^ calculated from the amino acid composition with ProtParam (https://web.expasy.org/protparam/ (accessed on 26 November 2023)) of 0.477 for wild-type *dh*PPase and most of its variants and 0.455 for the Y124A variant. Molar concentrations were calculated in terms of the subunit using a subunit molecular mass of 60.5 kDa.

P1,P4-Diadenosine 5´-polyphosphate (Ap_4_A, ammonium salt), AMP (free acid), ADP (di-monocyclohexylammonium salt), and ATP (di-sodium salt) were obtained from Sigma-Aldrich Co (St Lous, MO, USA). The concentrations of nucleotide stock solutions were estimated by measuring absorbance at 259 nm (ε = 15,400 M^−1^cm^−1^ for the mono-adenosine phosphates and 30,800 M^−1^cm^−1^ for Ap_4_A).

### 4.2. Enzyme Activity Assay

The initial rates of PP_i_ hydrolysis were measured using a continuous P_i_ assay [35]. The assay medium contained 0.1 M Mops-KOH, pH 7.2, 5.23 mM MgCl_2_, 140 μM PP_i_, (corresponding to 50 µM MgPP_i_ complex). Mg^2+^ complexation with AMP, ADP, and ATP was considered. The reaction was initiated by adding 0.1–10 nM enzyme and continued for 2–3 min at 25 °C. Rate values were obtained from the initial slopes of the P_i_ accumulation curves and were typically reproducible within 10%. Enzyme concentration in the assay was varied to obtain similar P_i_ accumulation rates in all cases, especially at low enzyme activities. Rate values (*v*) were subsequently normalized to the same enzyme concentration and weighed according to 1/*v*^2^ in the non-linear regression analysis.

### 4.3. Isothermal Titration Calorimetry (ITC)

Heat production upon nucleotide binding to dhPPase and its variants was measured at 25 °C using a VP-iTC calorimeter (MicroCal LLC., Northampton, MA, USA). Enzyme and adenine nucleotide solutions were prepared on 0.1 M Mops/KOH buffer (pH 7.2) containing 2 mM MgCl_2_, 0.1 mM CoCl_2_, and 150 mM KCl. Titrations were performed by successive 10-μL injections of 100–300 μM AMP, ADP, ATP, or 33–60 μM Ap_4_A solution into 1.4 mL of 8–10 μM protein solutions. The interval between injections was 5 min, and the injection time was 20 s. The measured heat values were corrected for ligand dilution effects. The ITC data were analyzed using a MicroCal ITC subroutine in Origin 7.0 (OriginLab Corporation, Northampton, MA, USA) using a single-binding-site model. The first 1–3 “anomalous” signals were usually discarded from this analysis.

### 4.4. Structure Modeling and Docking

The three-dimensional structure of the regulatory part of dhPPase (residues 68–303) was predicted from its amino acid sequence using AlphaFold2 (version 2.3.0) [36] with default parameter settings. All calculations were performed using an Nvidia RTX A5000 graphical card (Nvidia Corporate, Santa Clara, CA, USA). The twenty-five models generated (five models per prediction mod) were ranked according to their iptm + ptm score, and the best model (score = 0.899) was selected.

AMP molecules were docked into the modeled structure using AutoDock Vina program [37] (ver. 1.2.5.) with a grid size of 70 × 70 × 70 Å^3^. The twenty best models were selected for scoring in each calculation run. Final AMP positions agreed within 0.7 Å rmsd in three independent docking experiments. All structural visualizations were produced using UCSF Chimera (ver. 1.14) [38].

## Figures and Tables

**Table 1:** Parameters for tetramer ⇆ dimer equilibrium in *dh*PPase variants. Values of *k*_a_, *k*_d_, and their ratio, *K*_d_ = *k*_d_/*k*_a_, were estimated from inactivation time courses upon dilution (Figure 3) [11]. Zero-time wild-type *dh*PPase activity (*A*_T_ = 590 ± 30 U/mg) was taken as 100%. Effects greater than 10-fold are indicated in boldface.

Enzyme Variant	*A*_T_, %	*k*_d_, h^−1^	*k*_a_, μM^−1^·h^−1^	*K*_d_, μM
WT	100 ± 5	1.6 ± 0.1 (1.8 ± 0.1) ^a^	2.1 ± 0.5 (2.4 ± 0.4) ^a^	0.8 ± 0.2 (0.7 ± 0.2) ^a^
K95A	66 ± 2	1.00 ± 0.04	0.3 ± 0.1	3.2 ± 0.8
K100A	42 ± 1	1.1 ± 0.1	**0.19 ± 0.04**	5.9 ± 1.6
V117A	113 ± 1	0.44 ± 0.02	1.5 ± 0.2	0.3 ± 0.1
V117T	141 ± 5	0.6 ± 0.1	0.6 ± 0.3	1.0 ± 0.6
G118A	120 ± 3	1.06 ± 0.04	**0.15 ± 0.04**	6.8 ± 0.6
G118S	117 ± 2	0.85 ± 0.04	**0.14 ± 0.06**	6.0 ± 2.7
G118M	21 ± 1	0.70 ± 0.04	**0.18 ± 0.10**	4 ± 2
G118I	73 ± 1	0.75 ± 0.03	**0.12 ± 0.05**	6.2 ± 2.9
S121A	120 ± 3	1.5 ± 0.1	0.5 ± 0.1	3.3 ± 0.9
Y124A	83 ± 2	1.0 ± 0.1	0.9 ± 0.1	1.1 ± 0.2
E126A	82 ± 2	0.7 ± 0.1	3.4 ± 0.5	0.21 ± 0.05

^a^ Values in parentheses are from Anashkin et al. [11].

**Table 2:** Parameters of Equation (1) derived from the effects of the adenosine phosphates on the activities of 11 *dh*PPase variants (Figure 4).

Enzyme Variant	Adenosine Phosphate and Parameter Value ^a^											
	AMP			ADP			ATP			Ap_4_A		
	*A*_1_, %	*K*, µM	*h*	*A*_1_, %	*K*, µM	*h*	*A*_1_, %	*K*, µM	*h*	*A*_1_, %	*K*, nM	*h*
WT ^b^	3.7 ± 0.1	1.4 ± 0.1	1.7 ± 0.1	9 ± 1	0.95 ± 0.05	1.34 ± 0.03	250 ± 20	1.9 ± 0.2	1.45 ± 0.13	300 ± 10	4.9 ± 0.2	1.00 ± 0.02
K95A	1.4 ± 0.2	2.90 ± 0.04	1.7 ± 0.1	N.A. ^c^	N.A.	N.A.	317 ± 5	3.9 ± 0.1	1.63 ± 0.06	341 ± 6	10.9 ± 0.3	1.00 ± 0.02
K100A	2.3 ± 0.4	1.82 ± 0.02	**3.5 ± 0.1**	**750 ± 30**	**7.8 ± 0.2**	1.55 ± 0.05	**650 ± 20**	5.6 ± 0.1	1.26 ± 0.04	**1130 ± 20**	11.5 ± 0.1	1.07 ± 0.01
V117A	**66 ± 1**	**124 ± 5**	1.4 ± 0.1	**31 ± 1**	**13.4 ± 0.5**	1.60 ± 0.08	138 ± 1	0.95 ± 0.05	1.22 ± 0.07	145 ± 2	**120 ± 10**	1.06 ± 0.09
V117T	**46 ± 1**	**13.2 ± 0.4**	1.9 ± 0.1	**21 ± 1**	**7.9 ± 0.3**	1.48 ± 0.07	165 ± 4	**0.17 ± 0.02**	1.34 ± 0.12	202 ± 2	1.30 ± 0.05	1.02 ± 0.04
G118A	0.8 ± 0.7	8.4 ± 0.2	1.9 ± 0.1	N.A.	N.A.	N.A.	156 ± 2	2.00 ± 0.07	1.92 ± 0.12	219 ± 4	2.3 ± 0.2	1.02 ± 0.06
G118S	0.5 ± 0.6	2.55 ± 0.04	2.0 ± 0.1	N.A.	N.A.	N.A.	277 ± 5	0.82 ± 0.03	1.25 ± 0.05	312 ± 11	1.9 ± 0.2	1.03 ± 0.08
G118M	3.4 ± 0.6	0.76 ± 0.02	1.9 ± 0.1	N.A.	N.A.	N.A.	314 ± 8	4.9 ± 0.2	1.70 ± 0.12	**870 ± 30**	28 ± 1	0.99 ± 0.02
G118I	1.6 ± 1.0	2.90 ± 0.03	2.2 ± 0.1	240 ± 3	**17 ± 1**	1.5 ± 0.1	410 ± 12	6.2 ± 0.2	1.30 ± 0.06	393 ± 9	4.3 ± 0.2	1.05 ± 0.04
S121A	1 ± 4	1.60 ± 0.02	1.9 ± 0.1	4.2 ± 0.9	2.8 ± 0.1	2.0 ± 0.10	209 ± 2	1.27 ± 0.03	1.41 ± 0.05	247 ± 4	1.6 ± 0.1	0.97 ± 0.05
Y124A	2.6 ± 0.6	5.4 ± 0.1	1.6 ± 0.1	3.8 ± 0.6	5.0 ± 0.1	1.63 ± 0.04	299 ± 4	2.81 ± 0.06	1.40 ± 0.04	311 ± 7	1.8 ± 0.1	1.04 ± 0.05
E126A	0.2 ± 0.9	**22.5 ± 0.5**	1.8 ± 0.1	6.5 ± 1.6	**6.2 ± 0.3**	1.6 ± 0.1	172 ± 2	0.80 ± 0.06	1.25 ± 0.10	217 ± 4	2.9 ± 0.2	1.02 ± 0.07

^a^ Activity measured without adenosine phosphates (*A*_0_ in Equation (1)) was taken as 100% for each variant. Large effects are marked by boldface. ^b^ From Anashkin et al. [10] (Ap_4_A data) or from Anashkin et al. [9] (other adenosine phosphates). ^c^ N.A., not attendant.

**Table 3:** Parameters of Equation (2) for the four variants with the bell-shaped activity versus [ADP] profiles in Figure 4 ^a^.

Enzyme Variant	*A*_1_, %	*A*_2_, %	*K*_1_, μM	*K*_2_, μM	*h*_1_	*h*_2_
K95A	170 ± 30	18 ± 2	1.7 ± 0.6	7 ± 2	1.8 ± 0.3	1.4 ± 0.2
G118A	180 ± 20	91 ± 5	4.8 ± 1.5	50 ± 20	1.5 ± 0.2	1.3 ± 0.2
G118S	270 ± 30	0.8 ± 1.1	0.8 ± 0.2	3.0 ± 0.6	1.1 ± 0.1	1.1 ± 0.1
G118M	174 ± 19	10 ± 1	1.0 ± 0.5	10 ± 2	0.9 ± 0.1	0.9 ± 0.1

^a^ The activity measured without ADP (*A*_0_ in Equation (2)) was taken as 100% for each variant.

**Table 4:** The binding parameters derived from ITC titrations ^a^.

Enzyme Variant	Adenosine Phosphate	Δ*H*, kcal/mol	*n*	*K*_N_, µM	*−T*Δ*S*, kcal/mol
WT	AMP	−5.6 ± 0.5	0.79 ± 0.05	0.8 ± 0.3	−2.7 ± 0.9
K95A	AMP	−2.6 ± 0.2	**2.41 ± 0.04**	4.6 ± 0.7	−4.7 ± 0.4
K100A	AMP	−4.3 ± 0.1	**1.85 ± 0.04**	1.3 ± 0.2	−3.7 ± 0.3
V117A	AMP	−4.1 ± 0.6	0.94 ± 0.12	4.4 ± 0.8	−3.2 ± 0.8
V117T	AMP	−5.5 ± 0.2	0.94 ± 0.03	1.7 ± 0.2	−2.4 ± 0.3
G118A	AMP	−2.2 ± 0.1	**1.96 ± 0.09**	4.2 ± 0.6	−5.2 ± 0.2
G118S	AMP	−3.6 ± 0.2	1.16 ± 0.06	2.8 ± 0.4	−4.0 ± 0.3
G118M	AMP	−4.0 ± 0.1	0.98 ± 0.02	0.52 ± 0.07	−4.5 ± 0.2
G118I	AMP	4.6 ± 0.2	1.07 ± 0.03	1.6 ± 0.2	−3.3 ± 0.3
S121A	AMP	−5.1 ± 0.2	1.07 ± 0.02	1.1 ± 0.10	−3.1 ± 0.3
Y124A	AMP	−3.0 ± 0.2	1.10 ± 0.04	2.5 ± 0.2	−4.7 ± 0.2
E126A	AMP	−3.8 ± 0.2	1.05 ± 0.04	1.9 ± 0.4	−4.0 ± 0.4
WT	ADP	−5.9 ± 0.3	0.90 ± 0.04	1.0 ± 0.2	−2.3 ± 0.5
K95A	ADP	−3.6 ± 0.2	**2.1 ± 0.10**	9.3 ± 1.7	−3.2 ± 0.4
K100A	ADP	**−1.6 ± 0.2**	**2.1 ± 0.2**	6 ± 2	**−5.5 ± 0.5**
G118A	ADP	−3.3 ± 0.3	**2.1 ± 0.1**	5 ± 1	−4.0 ± 0.5
G118S	ADP	−5.8 ± 0.2	1.18 ± 0.03	1.1 ± 0.2	−2.3 ± 0.4
E126A	ADP	−5.9 ± 0.2	0.97 ± 0.02	0.6 ± 0.1	−2.6 ± 0.3
WT	ATP	−6.8 ± 0.2	0.88 ± 0.02	2.1 ± 0.3	−0.9 ± 0.3
K95A	ATP	−4.3 ± 0.7	**2.4 ± 0.3**	6 ± 2	−2.8 ± 1.0
K100A	ATP	**−0.41 ± 0.03**	**1.9 ± 0.1**	0.4 ± 0.2	**−8.3 ± 0.5**
G118A	ATP	**−1.3 ± 0.4**	**2.0 ± 0.3**	3 ± 4	**−6.3 ± 1.7**
G118S	ATP	**−1.0 ± 0.2**	1.1 ± 0.1	0.5 ± 0.5	**−8 ± 1**
E126A	ATP	−4.1 ± 0.6	1.0 ± 0.1	4.3 ± 1.2	−3.2 ± 0.9
WT	Ap_4_A	−10.3 ± 0.3	0.41 ± 0.01	N.D. ^b^	N.A. ^c^
K95A	Ap_4_A	−13.7 ± 0.6	0.45 ± 0.01	N.D.	N.A.
K100A	Ap_4_A	−11.4 ± 0.4	0.52 ± 0.01	N.D.	N.A.
V117A	Ap_4_A	−9.0 ± 0.3	0.41 ± 0.01	N.D.	N.A.
V117T	Ap_4_A	−9.1 ± 0.4	0.41 ± 0.01	N.D.	N.A.
G118A	Ap_4_A	−10.0 ± 0.2	0.47 ± 0.01	N.D.	N.A.
G118S	Ap_4_A	−8.0 ± 0.2	0.40 ± 0.01	N.D.	N.A.
G118M	Ap_4_A	−9.2 ± 0.4	0.45 ± 0.01	N.D.	N.A.
G118I	Ap_4_A	−10.0 ± 0.4	0.44 ± 0.01	N.D.	N.A.
S121A	Ap_4_A	−14.3 ± 0.3	0.42 ± 0.01	N.D.	N.A.
Y124A	Ap_4_A	−10.0 ± 0.4	0.41 ± 0.01	N.D.	N.A.
E126A	Ap_4_A	−10.7 ± 0.2	0.40 ± 0.01	N.D.	N.A.

^a^ Large effects are marked by boldface. ^b^ N.D., not determined. ^c^ N.A., not attendant.

**Table 5:** Summary of the effects of the substitutions in the CBS1 domain on the regulatory characteristics of CBS-PPase ^a^.

Residue	Direction of ADP Effect	Effect Size	Binding Stoichiometry	Binding Affinity	Binding Cooperativity	Tetramer Stability
Lys95	++		+++			− −
Lys100	+++	+++	+++		+++	− −
Val117	+	− − −		++		+
Gly118	+++	++	+	+	+++	− −
Ser121						−
Tyr124				+		
Glu126				+		+

^a^ The plus and minus signs indicate increase and decrease, respectively, in the numerical value of the characterizing parameter, where applicable, and their number indicates the relative size of the effect.

## Data Availability

Data are available on request from the corresponding author.

## Supplementary Materials

The supporting information can be downloaded at: https://www.mdpi.com/article/10.3390/ijms25115768/s1.
