# Supplementary material for: Specific Mutations Reverse Regulatory Effects of Adenosine Phosphates and Increase Their Binding Stoichiometry in CBS Domain-Containing Pyrophosphatase
Source: Int J Mol Sci. 2024 May 25;25(11):5768. doi: 10.3390/ijms25115768 (PMC11172384; doi:10.3390/ijms25115768)
Supplement: Supplementary file 1 [file ijms-25-05768-s001.zip › ijms-3006576-supplementary.pdf]

**Supplementary Material for:**

**"Specific Mutations Reverse Regulatory Effects of Adenosine Phosphates and Increase Their Binding Stoichiometry in CBS Domain-Containing Pyrophosphatase"**

Viktor A. Anashkin, Elena A. Kirillova, Victor N. Orlov, and Alexander A. Baykov

**Table S1.** The primers used for the site-directed mutagenesis of the CBS1 domain in *dh*PPase
The mutated nucleotides are shown in bold in the underlined codons

| *dh*PPase variant | Forward primer | Reverse primer |
|---|---|---|
| WT | TATACATATGTCAAAAAAAATCCATGTCG | TAATCTCGAGTTATTGAAGGAAATATTTC |
| K95A | TGGAAGATTATG**GC**AGAGAGTAACCA | TGGTTACTCTCT**GC**CATAATCTTCCA |
| K100A | GAGTAACCAA**GC**GACCCTGCCTG | CAGGCAGGGTC**GC**TTGGTTACTC |
| V117A | GATGATCACGG**C**AGGTGATTTATC | GATAAATCACCT**G**CCGTGATCATC |
| V117T | GGATGATCACG**ACC**GGTGATTTATC | GATAAATCACC**GGT**CGTGATCATCC |
| G118A | TGATCACGGTAG**C**TGATTTATCCG | CGGATAAATCA**G**CTACCGTGATCA |
| G118S | TGATCACGGTA**TC**TGATTTATCCGG | CCGGATAAATCA**GA**TACCGTGATCA |
| G118M | GATCACGGTA**ATG**GATTTATCCGG | CCGGATAAATC**CAT**TACCGTGATC |
| G118I | GATCACGGTA**AT**TGATTTATCCGG | CCGGATAAATCA**AT**TACCGTGATC |
| S121A | GGTAGGTGATTTA**GCT**GGTTCTTAC | GTAAGAACC**AGC**TAAATCACCTACC |
| Y124A | TCCGGTTCT**GCT**ATTGAGAGCATGG | CCATGCTCTCAAT**AGC**AGAACCGGA |
| E126A | GTTCTTACATT**GC**GAGCATGGCG | CGCCATGCTC**GC**AATGTAAGAAC |

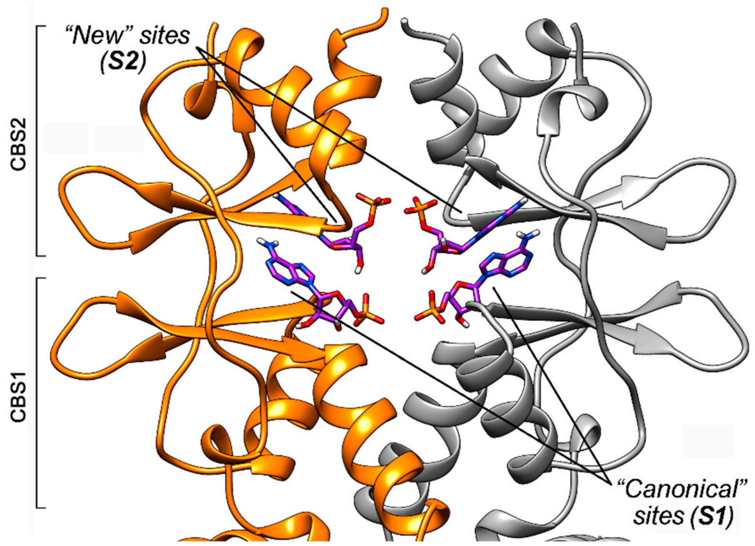


**Figure S1.** Docking of AMP into the modeled structure of the dimeric regulatory part of *dh*PPase K100A variant. The modeled structure comprising two CBS and one DRTGG domains (residues 68−303 of the complete protein sequence) was obtained using AlphaFold2 and is very similar to the crystal structure of the regulatory part of wild-type *cp*PPase presented in Figure 2 (rsmd of 1.3 Å for $C_\alpha$ atoms). AMP docking using AutoDock Vina detected two AMP-binding sites (S1 and S2) in each Bateman module with similar scores. Both sites locate between the CBS1 and CBS2 domains and are designated according to the CBS domain with which they form major interactions.
